# Hydroxychloroquine Does Not Function as a Direct Zinc Ionophore

**DOI:** 10.3390/pharmaceutics14050899

**Published:** 2022-04-20

**Authors:** Oisín N. Kavanagh, Shayon Bhattacharya, Luke Marchetti, Robert Elmes, Finbarr O’Sullivan, John P. Farragher, Shane Robinson, Damien Thompson, Gavin M. Walker

**Affiliations:** 1 SSPC, The Science Foundation Ireland Research Centre for Pharmaceuticals, V94 T9PX Limerick, Ireland; shayon.bhattacharya@ul.ie (S.B.); luke.marchetti@mu.ie (L.M.); robert.elmes@mu.ie (R.E.); finbarr.osullivan@dcu.ie (F.O.); johnpfarragher@gmail.com (J.P.F.); srobins2@its.jnj.com (S.R.); damien.thompson@ul.ie (D.T.); gavin.walker@ul.ie (G.M.W.); 2 School of Pharmacy, Newcastle University, Newcastle upon Tyne NE1 7RU, UK; 3 Department of Chemical Sciences, Bernal Institute, University of Limerick, V94 T9PX Limerick, Ireland; 4 Department of Chemistry, Maynooth University (National University of Ireland), W23 F2H6 Maynooth, Ireland; 5 National Institute for Cellular Biotechnology, Dublin City University, D09 NR58 Dublin, Ireland; 6 Department of Physics, Bernal Institute, University of Limerick, V94 T9PX Limerick, Ireland; 7 Janssen Pharmaceutical Sciences, T45 P663 Cork, Ireland

**Keywords:** hydroxychloroquine, COVID-19, ionophore, zinc, clioquinol

## Abstract

Drug-mediated correction of abnormal biological zinc homeostasis could provide new routes to treating neurodegeneration, cancer, and viral infections. Designing therapeutics to facilitate zinc transport intracellularly is hampered by inadequate concentrations of endogenous zinc, which is often protein-bound in vivo. We found strong evidence that hydroxychloroquine, a drug used to treat malaria and employed as a potential treatment for COVID-19, does not bind and transport zinc across biological membranes through ionophoric mechanisms, contrary to recent claims. In vitro complexation studies and liposomal transport assays are correlated with cellular zinc assays in A549 lung epithelial cells to confirm the indirect mechanism of hydroxychloroquine-mediated elevation in intracellular zinc without ionophorism. Molecular simulations show hydroxychloroquine-triggered helix perturbation in zinc-finger protein without zinc chelation, a potential alternative non-ionophoric mechanism.

## 1. Introduction

Ionophore molecules reversibly bind ions and facilitate their transport across cell membranes. Their ability to influence the disease state by altering intracellular ion concentrations creates new opportunities for pharmaceutics beyond traditional direct drug–protein targeting, with applications in Alzheimer’s disease (for which clioquinol and its derivatives have entered phase II clinical trials [1,2]) and cystic fibrosis (e.g., squaramides for chloride transport [3]), among many other potential treatments. The recent approval of the iron carrier (or siderophore) Cefiderocol [4] further demonstrates the utility of ionophores as molecular pharmaceutics.

Three key metrics characterize the ability of a molecule to function as a useful ionophore. First, the molecule must show significant complexation strength with the ion of interest; second, a certain degree of lipophilicity is required to transport the ion across membranes [5]; and third, to enable therapeutics, these binding and transport abilities must be replicated inside complex living systems such as cells.

At the beginning of the COVID-19 pandemic, clinicians had few therapeutic options, and as such, many efforts focused on repurposing of a wide variety of existing drugs. Claims were made at that time regarding the potential effectiveness of chloroquine and hydroxychloroquine (HCQ), and some studies suggested that zinc could be a valuable co-treatment option as it was postulated that HCQ can transport zinc to the site of action in the host cell through ionophorism [6]. Other authors suggested that co-administration of zinc and HCQ could increase intracellular zinc concentrations and inhibit RNA-dependent polymerase on which the virus depends for replication of its genetic code [6]. As HCQ was expected to behave in the same way as chloroquine–employing its aromatic nitrogen to chelate zinc [7]–and is the agent of choice in the clinic due to its favorable side-effect profile, there were a few limited clinical trials that co-administered zinc with HCQ (NCT04371406, NCT04334512, NCT04335084 and NCT04377646). Despite the growing knowledge and interest in biological zinc [8,9,10], little is known about structure-activity relationships of potential ionophoric compounds for it [11] and much of the literature explores the activity of well-known zinc ionophores, e.g., Clioquinol (CQ), pyrithione, and the 8-hydroxyquinoline analogue PBT2 [1,2,12,13,14].

**Scheme 1:**
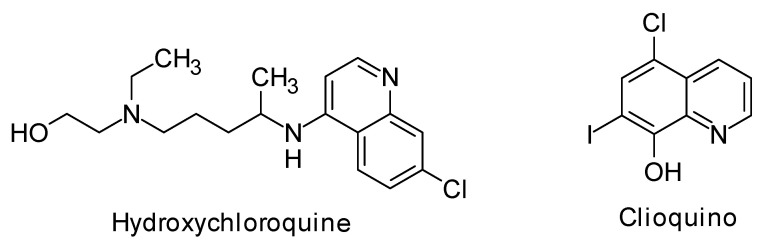
Structural comparison of hydroxychloroquine (HCQ) and clioquinol (CQ).

Here, we tested the postulated ionophoric mechanism of HCQ, seeking design rules for the rapid development of more effective zinc ionophores. Our study reveals that HCQ may not sequester zinc via traditional ionophoric mechanisms. CQ was employed for comparative purposes as a structural analogue of HCQ (Scheme 1) which has strong evidence of zinc ionophoric activity [13].

## 2. Results and Discussion

### 2.1. Quinoline–Zinc Structures in the Cambridge Structural Database

**Figure 1:**
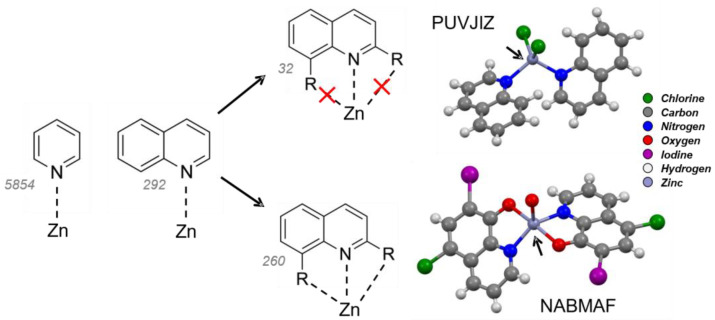
Cambridge Structural Database search for quinoline–zinc complexes with important structural features highlighted in two representative examples with CSD reference codes marked. Counts represent structures containing zinc based on the following criteria: contact inter/intra-molecular bonding (separated by 1–3 bonds 0.5–4.0 Å), R-factor < 0.05, non-disordered, no errors, single crystal structures and organometallic.

We surveyed the Cambridge Structural Database (CSD) (September 2021) to identify a total of 15,666 complexes containing zinc, where nitrogen was a common feature (n = 12,236/15,666). We noted that a significant fraction contained zinc bound to a pyridine group (n = 5854/12,236; see Figure 1), of which 292 involved quinoline functional groups. These functional groups (or synthons) identified in solid-state may also play a crucial role in their interactions with ions in solution. This group was further analyzed to quantify structures containing zinc bound directly to the quinoline with supporting groups on the same molecule (n = 260/292). The data suggests that suitably positioned electron donors on the quinoline molecule are important for binding zinc and that in CQ, zinc can form a 2:1 complex with zinc centered around the quinoline functionality (CSD reference code: NABMAF). In HCQ, zinc has been hypothesized to bind with weak affinity to the quinoline site (due to the lack of suitably positioned ancillary groups on the quinoline molecule) or to the ternary nitrogen [7,15,16]. There are no known structures of HCQ with zinc in the CSD although the pharmaceutical form hydroxychloroquine sulfate (CSD reference code: QOBHUL) is available. One structure illustrates the possibility of zinc complexation between two non-substituted quinoline groups (CSD reference code: PUVJIZ).

### 2.2. HCQ Does Not Exhibit Complexation Behavior with Zinc

Despite numerous reports of expected zinc binding by HCQ [6,14,17,18], there have been no further investigations to develop a structural understanding of these complexes. Previously reported studies indicate that chloroquine acts as a zinc ionophore [6], and HCQ would be expected to behave in the same way as chloroquine which uses its aromatic nitrogen to chelate to zinc [7].

**Figure 2:**
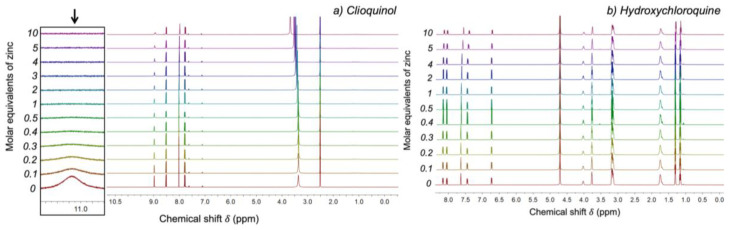
^1^H-NMR complexation studies of (**a**) CQ (in DMSO-d6) and (**b**) HCQ (in D_2_O) solutions titrated with ZnCl_2_ (in D_2_O).

NMR spectroscopy was used to test the hypothesis from CSD analysis that CQ uses the hydroxyl group on the quinoline ring to chelate zinc. This confirms that the quantity of hydroxyl protons decreases due to zinc binding (see Figure 2a). A slight shift across the whole spectrum was observed at the higher concentrations (Figure 2b), attributed to solvent effects [19].

**Figure 3:**
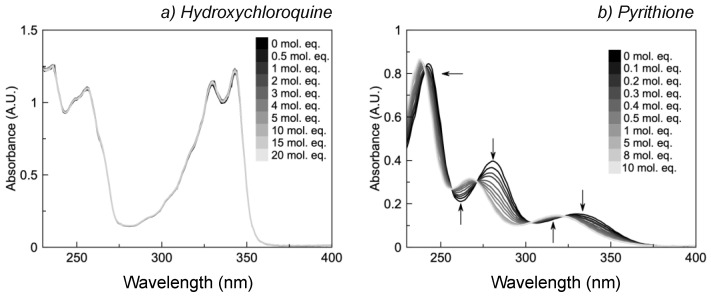
UV spectra of (**a**) HCQ and (**b**) pyrithione (5.01 × 10^−5^ M in Tris-buffered water) solutions titrated with aliquots of ZnCl_2_ in 0.1 molar equivalents. The final spectra for HCQ were 1:20 molar equivalents of drug to zinc.

**Figure 4:**
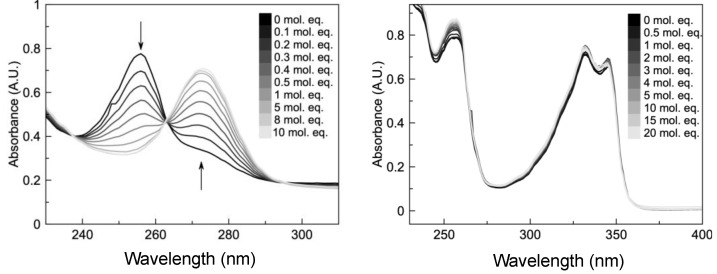
UV spectra of (**a**) clioquinol (CQ) and (**b**) hydroxychloroquine (HCQ) solutions (5.01 × 10^−5^ M in ethanol) titrated with aliquots of ZnCl_2_ in 0.1 molar equivalents. The final spectrum in each case (black line) was recorded for 1:2 (clioquinol) and 1:20 (hydroxychloroquine) molar equivalents of drug to zinc.

Extensive studies by Levy et al. [20] Higuchi et al. [21] and Connors et al. [22] have reported very low complexation constants for pharmaceuticals in water which are considerably higher in organic solvents. This understanding may be extended to ionophores where ion complexation to enable ionophoric capability [23] may be relatively low in the competitive high-dielectric aqueous environment and larger within non-polar cell membrane environments [24]. To investigate this, binding studies were carried out in Tris-buffered water, (Figure 3) and in ethanol (Figure 4) to simulate the change in lower dielectric conditions as the zinc complexes cross the membrane. To eliminate any potential interference of the buffer with complexation activity, further experiments were conducted in unbuffered water and HCQ (Figure S2) [22]. The experimental conditions were selected to reveal complexation activity—if any—and to emphasize that HCQ does not possess activity even at final concentrations twice that used for the positive control. Conversely, the conditions employed illustrate classic spectrometric changes that suggest complexation in pyrithione and clioquinol [22].

During the UV analysis in aqueous phase (Figure 3) and in ethanol (Figure 4, to emulate the serum and membrane environments, respectively), HCQ failed to demonstrate complexation of zinc up to 20 molar equivalents, in agreement with the NMR results above. Further experiments in unbuffered water demonstrate that HCQ does not exhibit a characteristic batho- or hypsochromic shift in saturated solutions of ZnCl_2_ (Figure S3). These studies indicate that HCQ does not complex with zinc in water at physiological pH, and nor does it complex with zinc in solvents that mimic the non-polar membrane environment. The confirmed ionophore behavior of clioquinol (CQ) provides an essential control to demonstrate the lack of ionophore activity of HCQ.

**Figure 5:**
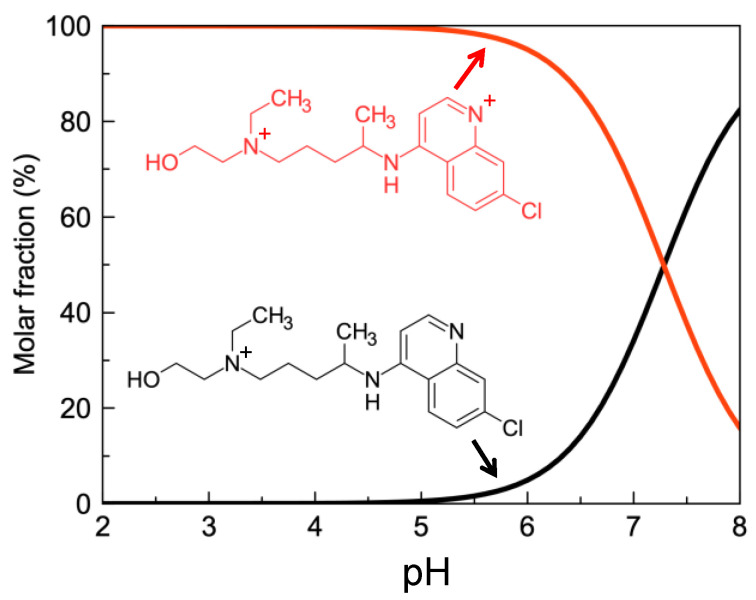
Bjerrum speciation plot for HCQ across the pH range encountered in vivo. Calculations performed using ChemAxon [25] which estimates a pK_a_ = 7.28 (quinoline) and 9.76 (ternary nitrogen) for HCQ.

As chemical speciation is known to have a dramatic effect on complexation, a Bjerrum plot to calculate the molar fraction of each HCQ species across the pH range encountered in vivo (Figure 5) reveals that there are two predominant states of HCQ. As all our experiments were conducted at pH 7.4, we expect approximately 50% of both species which suggests that zinc complexation of either of these HCQ species would have been observed, if feasible.

### 2.3. Liposomal Assays Confirm Lack of Ionophoric Activity by HCQ

**Figure 6:**
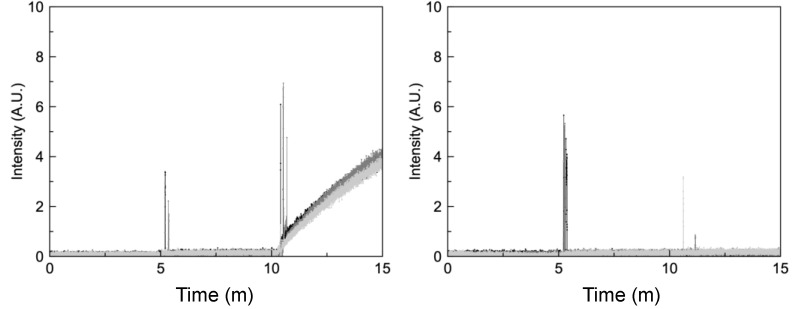
Fluorescence intensity of liposomal FluoZin-3 in PBS buffer (0.1 M, pH = 7.4) before and after addition of zinc and (**a**) clioquinol or (**b**) hydroxychloroquine at the ~10–11 min mark.

Liposomal assays have a long history as simple membrane mimetics [26] and can be used to evaluate ionophoric action as they can encapsulate ion selective molecular probes (e.g., FluoZin-3) to detect intraliposomal zinc. Various synthesis and purification techniques have emerged with extrusion methods producing liposomes of predictable polydispersity, which enables quantification of ionophore activity. To test if HCQ is an active ionophore in membrane environments, liposomal assays were conducted according to methods published by Jowett and Gale [14] (Figure 6). Figure S2 provides evidence that HCQ does not exhibit ionophoric behavior up to 90 min, in contrast to the immediate ionophoric behavior exhibited by CQ.

These results confirm the absence of zinc ionophoric activity by HCQ even in the presence of elevated zinc and HCQ concentrations (1 mM). By contrast, CQ demonstrates the typical expected ionophoric activity at 0.1 mM. There are some disadvantages with complexation and membrane penetration to determine the mechanism of reversible ion binding and transport by ionophore, despite being well-established techniques. In particular, the surface area available for diffusion and the lipid:ionophore concentration ratio may not be physiologically relevant, and the cell potential may underestimate the ionophoric action of molecules that act through symport or antiport mechanisms (i.e., moving pairs of ions across the membrane in the same or opposite direction) as the partner ions may not be present in the assay.

### 2.4. Cellular Toxicity and Fluorescence Imaging of HCQ Treated Cells

**Figure 7:**
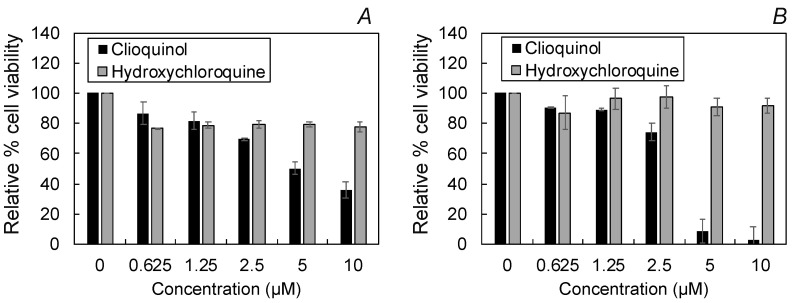
Cell viability after (**A**) 3 and (**B**) 7 days incubation with CQ and HCQ.

The evidence presented above suggests that HCQ may not operate as a direct ionophore. Cell toxicity assays were performed on the mammalian lung cell line A549 to determine the maximum concentrations of CQ and HCQ that could be tolerated by A549 cells without a large impact on cell viability (Figure 7). Our data showed no significant cell cytotoxicity up to 10 µM HCQ in 3- and 7-day toxicity assays.

These experiments revealed that at concentrations higher than 2.5 µM, cell viability was significantly reduced for CQ at both incubation times. Thus, 2.5 µM was selected as the concentrations of CQ and HCQ to be used in the zinc uptake cell assays (below). This value is consistent with reports that HCQ can inhibit viral spread in the Vero cell line (derived from African Green Monkey) [27] at EC_50_ values of 0.72–17.31 µM [28]. Furthermore, pharmacokinetic modelling predicted a mean plasma concentration of HCQ of 2.8 µM with common drug regimens, and other studies have concluded that approximately 3 µM serum HCQ concentration provides effective anti-rheumatic treatment [29].

**Figure 8:**
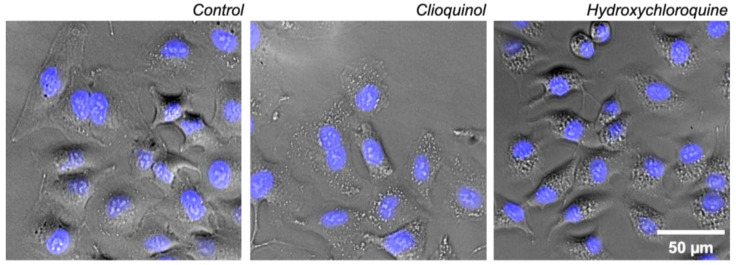
Cellular morphology of A549 control cell and cells treated for 24 h with 2.5 µM CQ or HCQ. Nuclei were counterstained with NucBlue Live to determine the number of nuclei and to assess gross cell morphology. Magnification ×20, scale bar 50 µm.

Morphological analysis by phase contrast microscopy revealed a pronounced increase in intracellular vesicles after 24 h treatment with 2.5 µM HCQ (Figure 8). The formation of these vesicles may potentially indicate that cells are responding to increased intracellular zinc by forming discrete vesicles known as zincosomes [30] or that the drug molecules are being localized in a vesicle compartment [12,31,32,33,34]. The cell morphologies of the treated cells are consistent with this hypothesis, showing a marked difference in the granular appearance of the cells treated with HCQ.

**Figure 9:**
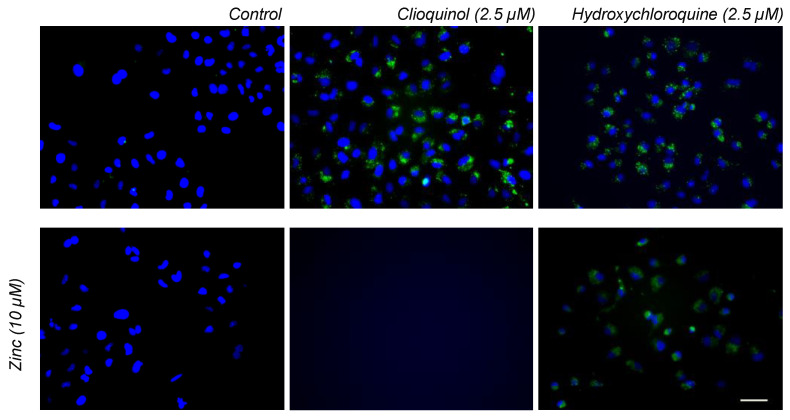
Fluorescence images of A549 cells stained with FluoZin-3 AM (green) and counterstained using NucBlue Live for nuclei (blue) after 24 h incubation with CQ or HCQ and with basal and additional 10 µM zinc. Magnification ×10, scale bar 50 µm.

The cell morphology data were complemented by fluorescence imaging using a zinc-sensitive fluorescent stain FluoZin-3 (Figure 9). Control experiments were performed without additional zinc to mimic the physiological conditions. Cells were cultured in basal media supplemented with serum which provided cells with sufficient zinc for cellular requirements. Furthermore, the same batch of media and serum were used throughout to minimize artefacts from other trace metal elements [35].

The ability of 2.5 µM CQ and HCQ to increase intracellular zinc compared to control after 24 h incubation was assessed using the fluorescent dye FluoZin-3 AM. Both CQ and HCQ show increased FluoZin-3 AM staining compared to the control which appeared as discrete vesicle staining. These results confirm the morphological analysis, suggesting that zinc localizes within zincosomes. Alternative zinc measurements, such as ICP-MS or using a different zinc fluorescent indicator, could be used in future studies to further explore our observed HCQ-induced cellular zinc accumulation. The staining patterns for both compounds were similar to basal Zn levels. Co-incubating with additional 10 µM zinc in the form of ZnCl_2_ resulted in complete cell death for CQ. By contrast, the additional zinc potentiates HCQ toxicity (similar to previous reports using CQ [6]) but the lack of complete cell kill suggests that HCQ is not as sensitive as CQ to extracellular zinc concentrations. Sensitivity to extracellular zinc is an important characteristic one would expect from an ionophore, which further points to a lack of direct ionophore action by Zn. The ionophoric mechanism of CQ allows Zn in the basal media to cross the cell membrane. As ionophores are expected to function along a concentration gradient, one might expect increased toxicity when the cells are incubated with additional Zn, which is demonstrated with CQ. By contrast, HCQ does not behave in this way, and there is little evidence of increased toxicity. This suggests that although HCQ appears to increase intracellular zinc, it is not through ionophoric mechanisms.

### 2.5. Molecular Models Predict Non-Ionophoric Mechanism for Future Study

**Figure 10:**
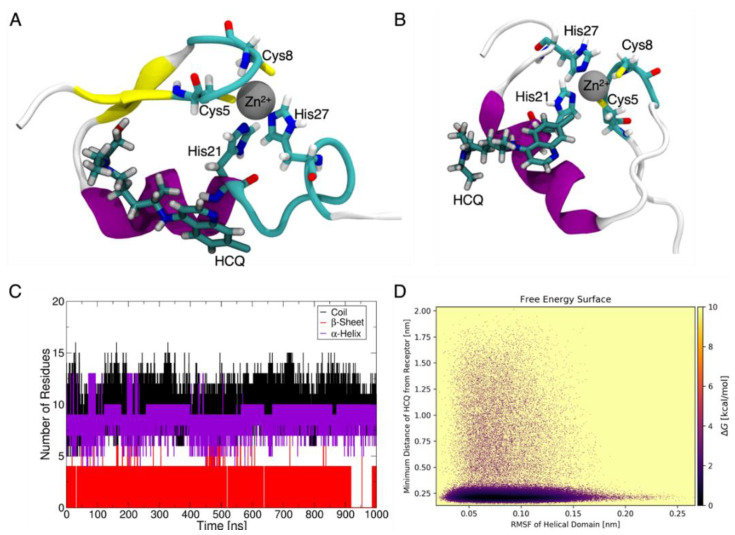
Representative structures of HCQ-bound zinc-finger motif showing (**A**) HCQ disrupting the 𝛼-helical domain during the first 500 ns of room temperature dynamics in water, and (**B**) HCQ interacting with Zn^2+^ ion causing temporary unfolding of the *β*-sheet domain during the second 500 ns of dynamics. (**C**) Timeline of number of residues in coil, *β*-sheet and 𝛼-helix conformation for the full microsecond dynamics of zinc-finger motif with HCQ. (**D**) Free energy map of HCQ–zinc-finger distance against root mean square fluctuation (RMSF) of the 𝛼-helical domain showing exclusively large RMSF only with bound HCQ.

Finally, we report below molecular dynamics models that predict a significant perturbation of the 𝛼-helical fold of the zinc-finger Zn-storage protein in the presence of HCQ (Figure 10). The models show that HCQ engages in only weak, non-specific binding to the finger, causing no significant disruption to Zn binding during the microsecond timescale of the simulations in water (see Supplementary Information, Figures S4–S6). Figure 10A shows a representative structure of HCQ interaction with the finger with loss of the 𝛼-helical fold within the first 0.5 microseconds, while during the second 0.5 microseconds, the *β*-sheet becomes unstable and is temporarily lost (precisely after 0.92 microseconds; see Figure 10C) due to perturbation by HCQ (see the snapshot of a representative HCQ:Zn binding event in Figure 10B). The corresponding free energy maps reveal that the helix undergoes significant fluctuations and disordering only when HCQ is bound (Figure 10D) to the finger, during room temperature dynamics in water. This potential helix perturbation by HCQ predicted by the molecular models suggests a possible avenue for future experiments targeting Zn-storage proteins as a potential source of elevated intracellular zinc in the presence of HCQ and other non-ionophoric drugs. Direct experimental measurement via structural elucidation will be essential to establish the details of alternative non-ionophoric mechanisms the molecular models presented here are speculative and are included simply to visualize what a potential non-ionophoric mechanism may involve. The details are beyond the scope of this first report that HCQ does not act as a direct zinc ionophore. In the future, protein crystallography experiments involving challenging crystallization experiments and use of synchrotron radiation may provide high-resolution Zn-protein complexes to further delineate possible alternative mechanisms.

## 3. Conclusions

Based on a large dataset obtained from experiments ranging from molecular complexation studies to cell assays, we conclude that HCQ does not function as a direct zinc ionophore, in stark contrast to the CQ control. Ionophores are an exciting new class of molecular pharmaceutics, but they have a very clear mechanistic definition. Mislabeling these mechanisms could delay translation of valuable therapeutics, by complicating the interpretation of pre-clinical and clinical results. Using cellular assays within the context of complexation and liposomal assays may serve as a useful test of ionophoric mechanisms [36].

Future work in this field may include an analysis of ion transport facilitated by other existing and potential drugs. The demonstrated non-conventional behavior of HCQ provides a new opportunity to increase the net intracellular flux of metals through potentially uncharacterized drug-action mechanisms, which could include the potential perturbation of metal storage enzymes, participation of metal transport proteins on cell surfaces or metal release coupled with drug binding dynamics within vesicles, among other tantalizing possibilities.

## 4. Materials and Methods

This study was conducted concurrently with another [36] and as such many of the methods and techniques employed in this study are similar. Assoc. Prof. Lidia Tajiber (Trinity College Dublin) donated hydroxychloroquine sulfate, its purity was confirmed by ^1^H-NMR (Figure S1). Clioquinol was purchased from Tokyo Chemical Industries and cholesterol and zinc chloride were purchased from FluoroChem. The extrusion kit, and materials required to create FluoZin-3 loaded liposomes i.e., 0.2 µm polycarbonate membrane filters, FluoZin-3 (membrane impermeable) and 1-palmitoyl-2-oleoyl-glycero-3-phosphocholine (POPC) were purchased from Avanti Polar lipids. All other solvents and materials were purchased from Sigma-Aldrich at the highest grade available.

### 4.1. Cambridge Structural Database

The Cambridge Structural Database was searched using ConQuest (v5.40, April 2021), and the retrieved entries were subsequently analyzed with Mercury. Structures containing zinc here selected based on the following criteria: contact inter/intra-molecular bonding (separated by 1–3 bonds 0.5–4.0 Å), R-factor < 0.05, non-disordered, no errors, single crystal structures and organometallic.

### 4.2. Complexation Studies

Stock solutions (5 × 10^−^^6^ mM) of hydroxychloroquine sulfate, zinc and pyrithione were prepared using deionized water and aliquoted to a 3 mL solution of trisaminomethane (Tris, 0.01 M, at pH 7.4). Concentrations of hydroxychloroquine and pyrithione used were determined by preliminary experiments to obtain an absorbance of approximately below 1 A.U. These solutions were then titrated with aliquots of ZnCl_2_ in 0.05 molar equivalents and absorbance was recorded in a quartz glass cuvette (1 cm pathlength) from 230 to 400 nm. Each solution was prepared freshly prior to every experiment and this procedure was repeated in triplicate.

For NMR complexation studies, a 5.0 × 10^−5^ M solution of hydroxychloroquine sulfate or clioquinol was prepared in D_2_O and DMSO-d6, respectively, and titrated with 5.0 × 10^−6^ M ZnCl_2_ prepared in D_2_O. Different solvent systems were used for clioquinol and hydroxychloroquine due to precipitation occurring if the same solvent system was used (i.e., clioquinol precipitates from D_2_O and hydroxychloroquine precipitates from DMSO-d6). As such, the goal of NMR complexation studies in this instance was to confirm the functional groups involved in clioquinol complexation to zinc.

### 4.3. Synthesis of FluoZin-3 Loaded Liposomes

Liposomes were synthesized according to a previously reported procedure [14]. POPC (250 mg, 3.289 × 10^−4^ moles) and cholesterol (1.4125 mg, 3.65 × 10^−^^6^ moles) were dissolved in 8 mL chloroform and 4 mL of this solution was aliquoted into a 25 mL Round Bottom Flask (RBF). The solvent was then removed by rotary evaporation to obtain a thin film and 0.5 mL DMSO containing 590.3 µM of FluoZin-3 was mixed with 3.5 mL PBS buffer (0.01 M PBS, at pH 7.4), added to the RBF, and subsequently vortexed for 15 min. This mixture was then freeze-thawed 15 times and subsequently passed through a Sephadex G100 column (previously swelled for 24 h with PBS) to remove any unencapsulated FluoZin-3 from the solution.

This liposomal solution was extruded 25 times through a 0.2 µm polycarbonate membrane using an Avanti extrusion kit and then stored in a screwcap glass vial covered with tinfoil and kept in the fridge (2–8 °C) until use.

### 4.4. Liposomal Penetration Assay

Stock solutions of liposomes, clioquinol or hydroxychloroquine and zinc were prepared and allotted made up to 2.5 mL in PBS buffer (0.01 M PBS, at pH 7.4) to create a final solution containing 52 µM lipid to 100 mM of ionophore and zinc (1000 mM was used in the case of HCQ). Fluorescence was determined using a quartz cuvette in a Varian Eclipse fluorospectrometer exciting and emitting at 494 nm and 519 nm, slits were set to 5 nm. During the experiment, the fluorescence measurement was started for 5 min to obtain a baseline. Then zinc was added followed by either hydroxychloroquine or clioquinol after 5 min and the fluorescence intensity was recorded for a further 5 min.

### 4.5. Cell Culture Conditions

Exponentially growing type II lung epithelial cells (A549 ATCC^®^ CCL-185) were cultivated at 37 °C under 5% CO2, in 1:1 DMEM:F-12 medium (Sigma-Aldrich D8437, Darmstadt, Germany) supplemented with 10% fetal bovine serum (FBS, Gibco, 10270-106, Thermo Fisher Scientific, Waltham, MA, USA). Cells were passaged approximately every 3–4 days, to prevent cells from reaching confluence. Subculture was carried out by first washing the cell monolayers with phosphate buffered saline (PBS) followed by incubation with TrypLE Trypsin-EDTA (0.05%, Gibco, 25300-062, Thermo Fisher Scientific, Waltham, MA, USA) at 37 °C as above. Cell number and viability were determined by staining with trypan blue and counting with a hemocytometer.

### 4.6. Cell Toxicity Assay

A549 cells were plated at a density of 1 × 10^2^ cells per well (for 7 days) and at 1.4 × 10^2^ cells per well (3 days). After allowing 24 h for attachment, clioquinol and hydroxychloroquine were aliquoted to the wells to reach final concentrations of 10, 5, 2.5, 1.25 and 0.625 µM and incubated for 3 and 7 days. Cell viability was assessed by incubating with Alamar Blue HS (Invitrogen A50101, Thermo Fisher Scientific, Waltham, MA, USA) After incubation, 20 µL of Alamar Blue (final concentration 10% *v*/*v*) was added to each well and the plates were further incubated for 2 h. Fluorescence intensity was recorded with excitation at 530 nm and emission at 590 nm. This procedure was repeated to generate three biological repeats with a minimum of six technical repeats in each set.

### 4.7. Fluoresczence Imaging

A549 cells were plated in a 24 well plate at a density of 3 × 10^4^ per well. After allowing 24 h for attachment, clioquinol and hydroxychloroquine were added to the wells to give a final concentration of 2.5 µM and further incubated for 24 h. The wells were then washed with PBS and stained with FluoZin-3 AM (Invitrogen F24195, Thermo Fisher Scientific, Waltham, MA, USA) and Pluronic-24 (final concentration of FluoZin-3 AM was 1 µM) nuclei were counterstained with NucBlue Live (Invitrogen R37605) and further incubated for 1 h. The cells there washed again with PBS and 250 µL serum free media (DMEM:F12) was added to each well before imaging. Cells were imaged using a Nikon TiE equipped with Photometrics Coolsnap HQ2 camera () and controlled by Metamorph. Zinc influx by FluoZin-3 has been demonstrated to be selective and sensitive for zinc across studies conducted in several labs, cell types and experimental methods [13].

### 4.8. Molecular Dynamics Simulations and Supplementary Analyses

A high-resolution solution NMR structure of zinc-finger protein with PDB code 3ZNF [37] was used as the starting structure for microsecond atomistic molecular dynamics (MD) simulations in water at room temperature. The zinc-finger has two anti-parallel 𝛽-strands (residues 3–4 and 9–12) forming 𝛽-sheet connected by an atypical turn (residues 3–12) containing the Zn^2+^-coordinating residues Cys5 and Cys8 and an 𝛼-helix (residues 14–24) which houses the His21 coordinating residue, while the second histidine, His27 lies in the C-terminus loop (residues 25–30). The starting structure of HCQ-bound zinc-finger was created by manually placing the drug HCQ in the groove adjacent to the Zn binding site (see Figure S4B). As a control, we also performed MD runs on the zinc-finger protein only, without HCQ (see Figure S4A). Both systems were sampled for 1 microsecond of free dynamics. CHARMM36m forcefield parameters [38] were used to describe the zinc-finger protein atoms and Zn metal ion. The HCQ molecule was parametrized with ParamChem [39,40], which provides the complementary CHARMM General Force Field (CGenFF) [41] parameters. Additionally, the partial charges on the drug molecule atoms were derived using the Restrained Electrostatic Potential (RESP) [42] scheme based on quantum mechanical calculations with Gaussian 16 [43], followed by charge fitting with Antechamber [44]. CHARMM-modified TIP3P [45] was used to model the water and a minimum distance of 20 Å was maintained between any protein atom and any edge of the simulation box. The MD simulations were carried out using the Gromacs 2018.4 [46,47] package with an integration time step of 2 fs implemented in the leapfrog integrator [48] with bond lengths to hydrogen constrained using the LINCS [49] (protein) and the SETTLE [50] (water) algorithms. Snapshots were saved every 2 ps. Background ions were added to neutralize protein formal charges and to model physiological ionic strength (0.15 M NaCl). Periodic boundary conditions were applied to mimic bulk solvation with long-range electrostatics treated by the Particle Mesh Ewald (PME) method [51]. Protein and non-protein molecules (water and ions) were coupled separately to an external heat bath (310 K) with a coupling time constant of 1 ps using the velocity rescaling method [52]. All systems were energy minimized, and thermalized over 100 ps, and equilibrated for 1 ns in constant volume NVT ensemble followed by another 1 ns of NPT equilibration with the reference pressure at 1 bar and a time constant of 4 ps using the Berendsen barostat [53]. The production runs were carried out in the constant pressure NPT ensemble using the Parrinello–Rahman barostat [54].

## Data Availability

Data available on request.

## Supplementary Materials

The following supporting information can be downloaded at: https://www.mdpi.com/article/10.3390/pharmaceutics14050899/s1, Figure S1: H^1^-NMR oh hydroxychloroquine sulfate in D_2_O to confirm its identity and purity; Figure S2: UV-Vis spectra of HCQ and HCQ in unbuffered (Type II deionized water) and saturated zinc chloride aqueous solutions; Figure S3: Preliminary liposomal assay monitoring fluorescence intensity of liposomal FluoZin-3 in PBS (0.1 M, pH = 7.4) before and after addition of zinc and clioquinol or hydroxychloroquine. Ionophore was added to solutions at 30 min; Figure S4: Initial (**A**,**B**) and final (**C**,**D**) structures from 1 microsecond dynamics of zinc-finger protein alone (**A**,**C**) and in the presence of HCQ (**B**,**D**); Figure S5: Timelines of interaction energies between the zinc ion and its coordinating residues (**A**) without HCQ and (**C**) with HCQ. Timelines of minimum distances of zinc ion from its coordinating atoms (**B**) without HCQ and (**D**) with HCQ. Protein—Zn coordination sphere structure, dynamics, and binding energetics are statistically indistinguishable with and without HCQ; Figure S6: Simulation timelines of (**A**) HCQ–protein interaction energies and (**B**) minimum distances of any atom of HCQ from the protein (black) and zinc ion (red).
