# Supplementary material for: Hydroxychloroquine Does Not Function as a Direct Zinc Ionophore
Source: Pharmaceutics. 2022 Apr 20;14(5):899. doi: 10.3390/pharmaceutics14050899 (PMC9147311; doi:10.3390/pharmaceutics14050899)
Supplement: Supplementary file 1 [file pharmaceutics-14-00899-s001.zip › pharmaceutics-1665057-supplementary.pdf]

Supplementary information

Hydroxychloroquine Does Not Function as a Direct Zinc Ionophore

Oisín N. Kavanagh [1,2,3,4,5,*], Shayon Bhattacharya [1,6], Luke Marchetti [1,4], Robert Elmes [1,4], Finbarr O'Sullivan [1,5], John P. Farragher [1], Shane Robinson [1,7], Damien Thompson [1,6] and Gavin M. Walker[1,3]

Supplementary information

H[1]-NMR confirms the presence and purity of HCQ. All protons are characterized in this figure and notably there is no signal at 11 ppm as it does not contain the hydroxyl group on the quinoline ring.

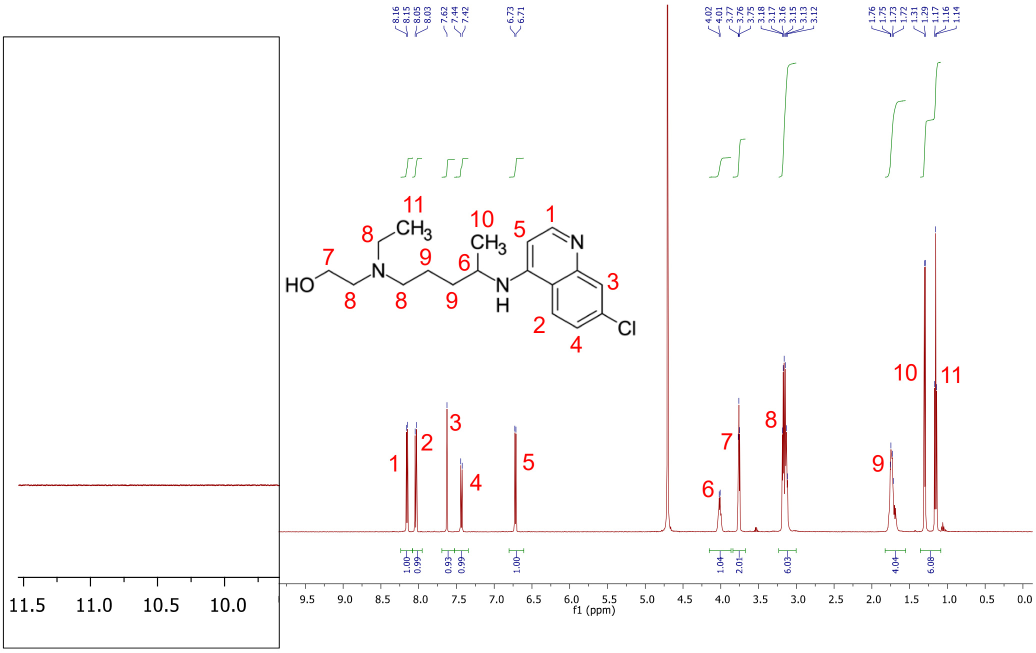

Figure S1. H[1]-NMR oh hydroxychloroquine sulfate in D_2O to confirm its identity and purity.

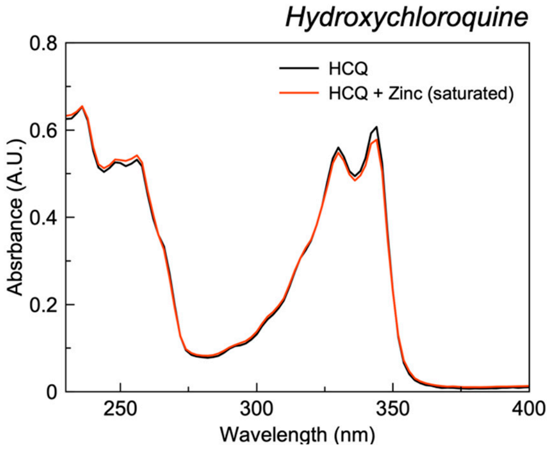

Figure S2. UV-Vis spectra of HCQ and HCQ in unbuffered (Type II deionized water) and saturated zinc chloride aqueous solutions.

Preliminary experiments illustrated that ionophoric activity is almost instant and that HCQ has no ionophoric activity up to 90 min, while clioquinol activity is almost instant (Figure S3).

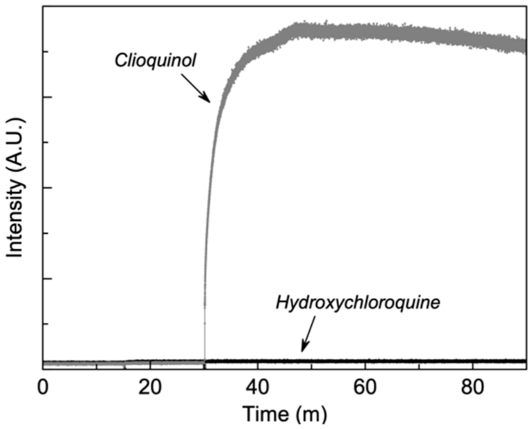

Figure S3. Preliminary liposomal assay monitoring fluorescence intensity of liposomal FluoZin-3 in PBS (0.1 M, pH = 7.4) before and after addition of zinc and clioquinol or hydroxychloroquine. Ionophore was added to solutions at 30 min.

The computed molecular dynamics shows that the zinc-finger binding site residues (two Cys and two His) firmly hold the Zn^{2+} ion in place (Figure S4C), while the HCQ samples a multitude of binding modes on the zinc-finger that do not directly loosen Zn^{2+} (Figures S4 and S5) but trigger subtle structural changes to the protein (main text Figures 10 and S6). To characterize these potential HCQ-triggered changes to the Zn finger, we mapped timelines of secondary structure elements of the zinc-finger protein. We observe that the α-helical region (residues 14–24) becomes unstable during the first 500 ns of dynamics (Figure 10C) and the protein loses its β-sheet content (residues 3–4 and 9–12) towards the end of dynamics (Figure 10C). The free energy map in Figure 10D shows that the perturbation in the α-helix is associated exclusively with close proximity of HCQ, and we also see more direct HCQ contacts with the zinc ion itself during the last 200 ns (Figure S6), which do not directly weaken the Zn coordination sphere (Figure S5) but precede loss of the β-sheet motif of the finger (Figure 10C).

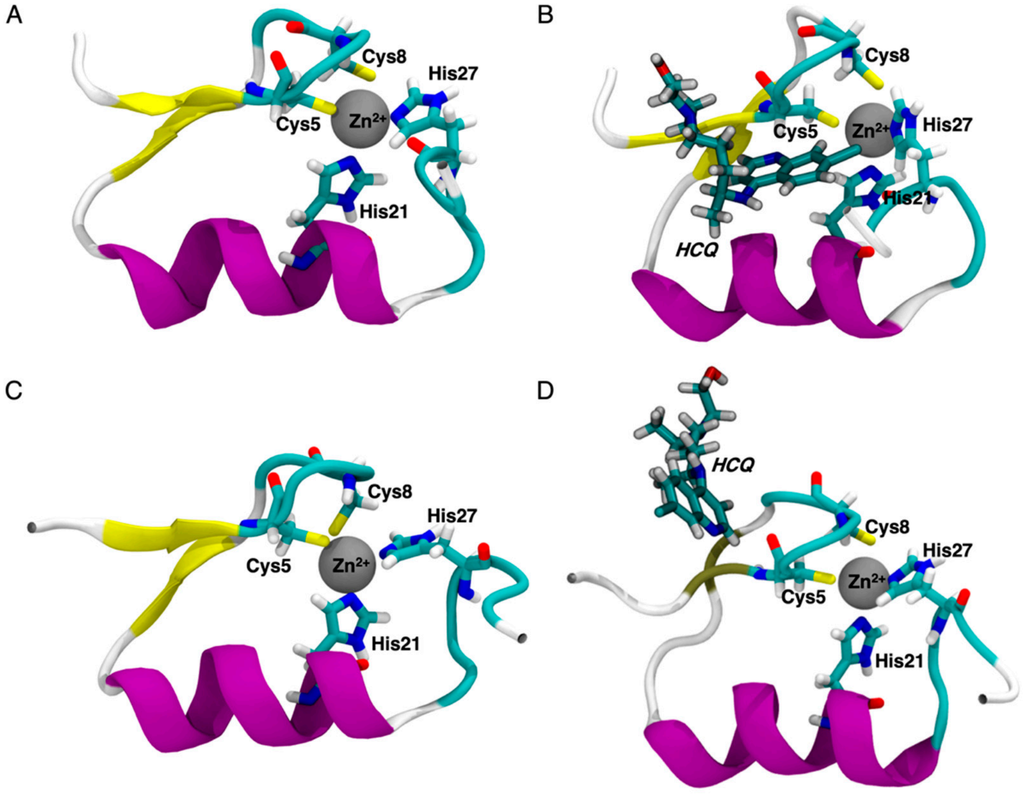

Figure S4. Initial (**A**, **B**) and final (**C**, **D**) structures from 1 microsecond dynamics of zinc-finger protein alone (**A**, **C**) and in the presence of HCQ (**B**, **D**).

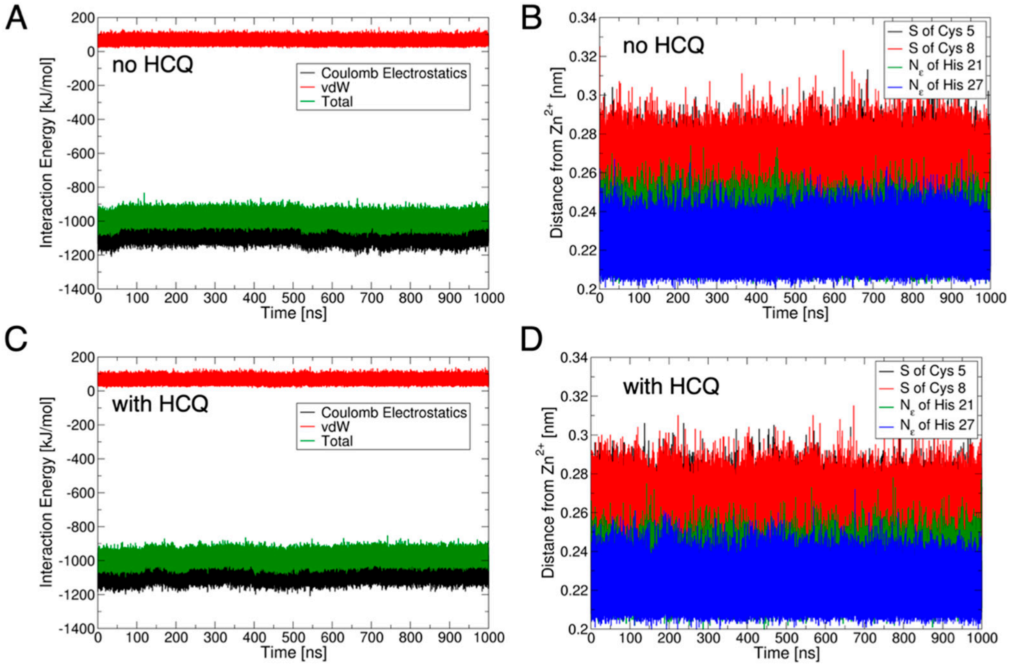

Figure S5. Timelines of interaction energies between the zinc ion and its coordinating residues (**A**) without HCQ and (**C**) with HCQ. Timelines of minimum distances of zinc ion from its coordinating atoms (**B**) without HCQ and

(**D**) with HCQ. Protein—Zn coordination sphere structure, dynamics, and binding energetics are statistically indistinguishable with and without HCQ.

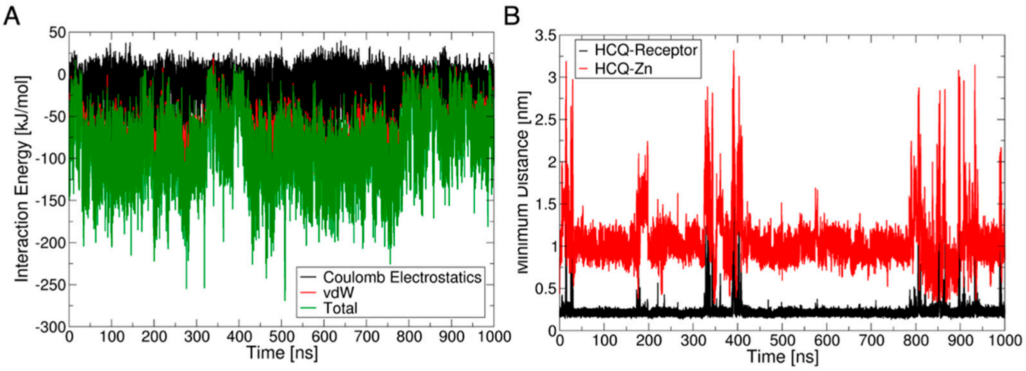

Figure S6. Simulation timelines of (**A**) HCQ–protein interaction energies and (**B**) minimum distances of any atom of HCQ from the protein (black) and zinc ion (red).
